# The moderating effect of attitudes in the relationship between knowledge and self-efficacy in palliative care among nurses: A cross-sectional, correlational study

**DOI:** 10.1371/journal.pone.0292135

**Published:** 2023-10-05

**Authors:** JinShil Kim, Seongkum Heo, Jisun Yang, Miyeong Kim, SeongHu Park, KyungAh Cho, JungHee Kang, Hani Yi, Minjeong An

**Affiliations:** 1 College of Nursing, Gachon University, Incheon, South Korea; 2 Georgia Baptist College of Nursing, Mercer University, Atlanta, GA, United States of America; 3 Department of Nursing, Gachon University Gil Medical Center, Incheon, Korea; 4 College of Nursing Sciences, Sungshin Women’s University, Seoul, South Korea; 5 College of Nursing, University of Kentucky, Lexington, Kentucky, United States of America; 6 Department of Nursing, Asan Medical Center, Seoul, South Korea; 7 College of Nursing, Chonnam National University, Gwangju, South Korea; Ajou University, REPUBLIC OF KOREA

## Abstract

Provision of palliative care to patients with advanced chronic diseases or old populations is suboptimal, which results in unnecessary suffering of and burden to patients, caregivers, and society. Low self-efficacy in palliative care among nurses is a factor affecting suboptimal utilization of palliative care. Poor knowledge is a factor affecting low self-efficacy in palliative care of nurses. Attitudes may contribute to the relationship between knowledge and self-efficacy in palliative care, but these relationships have been rarely examined in nurses. This study aimed to determine whether nurses’ attitudes moderate the relationship between knowledge and self-efficacy in palliative care. In a cross-sectional, correlational study, online or offline survey on self-efficacy, knowledge, attitudes, and covariates was conducted from 282 nurses in South Korea. PROCESS v4.1 for SPSS was used to address the study aim. Higher levels of knowledge (p = .048) and attitudes (p < .001), and the interaction term of knowledge and attitudes (p = .025) were significantly associated with higher levels of self-efficacy (F = 6.12, p < .001, R^2^ = .152), indicating the moderating effects of attitudes. The relationships between higher levels of knowledge and self-efficacy were significant only in nurses with highly and moderately positive attitudes (R^2^ change = .016, F = 5.11, p = .025), but not nurses with lack of positive attitudes. Our results supported the moderating role of nurses’ attitudes in the relationship between knowledge and self-efficacy. To improve self-efficacy in palliative care in nurses, improvement in knowledge and facilitation of positive attitudes are needed.

## Introduction

Early discussion about end-of-life (EOL) is highly recommended in many clinical contexts [1–5]. By integrating such discussion into the routine care, a periodic and ongoing EOL discussion may occur through advance care planning (ACP) [3]. In ACP process, an advance directive (AD) can be signed that enables an individual to prepare future palliative and EOL care based upon own personal values and preferences [3, 4, 6–8]. The planned care reduces personal and societal burdens of care and improves quality of EOL care by reflecting personal desires [8–10], more completing an AD and /or documentation of ACP [8–10], and less using resources with lower costs during the EOL period [8, 11]. Conversely, suboptimal utilization of EOL discussion via ACP and/or ADs often results in patients receiving meaningless aggressive treatments until near death [12] and EOL care against their preferences [8, 13, 14]. Therefore, utilization of EOL discussion needs to be facilitated.

Nurses can initiate or facilitate such EOL discussion by assisting an individual and his/her family to make informed and shared decision for future medical care and eventually can provide the EOL care in the future [4, 15–18]. However, engagement in EOL care in healthcare providers, including nurses, was still low [19]. As a key stakeholder who provides palliative care, the likelihood of nurses’ engagement in EOL care increases with high levels of self-efficacy in palliative care or ACP [20–23]. Self-efficacy in palliative care of nurses may empower nurses to actively engage in EOL discussion and to demonstrate their competence in managing and assisting an individual and family member with various needs and decisions for future care at the EOL [20–23]. However, self-efficacy in palliative care or ACP in nurses is moderately low [24]. Especially, because nurses are key providers who are managing a wide spectrum of EOL needs in individuals and their families, increasing nurses’ self-efficacy in counseling and initiating the EOL discussion is important [25].

There are some known factors affecting self-efficacy in palliative care or ACP in healthcare providers. Knowledge deficit and negative attitudes toward the EOL issues, including ACP and ADs, and related legal and ethical issues are factors affecting self-efficacy in nurses [21, 24, 26]. Oncology nurses showed a low level of self-efficacy toward palliative care, and lower palliative care knowledge and duration of oncology nursing practice significantly predicted lower levels of self-efficacy [26]. In another study [24], healthcare providers (doctors and nurses who primarily provided care to the patients with non-malignancy, chronic diseases) showed a moderately low level of self-efficacy, and lower levels of knowledge and less positive attitudes were associated with lower levels of self-efficacy. On the other hand, in a sample of intensive care nurses with more than 15 years of working experiences in approximately one-third (36.2%), a lack of knowledge in the majority (81.1%), and a high level of self-efficacy in more than a half (56.7%), higher levels of knowledge and more positive perceptions related to palliative care were associated with higher levels of self-efficacy [21].

In addition to the relationship between attitudes and self-efficacy, attitudes were also associated with knowledge in nurses who caring non-cancer patients (e.g., heart failure, stroke, or other organ failures, i.e., kidney and liver) [27]. Thus, attitudes toward ADs may have a moderator role in the relationship between knowledge and self-efficacy in palliative care. However, the relationships have been rarely examined in nurses. Examination of the mechanism of the relationships among knowledge, attitudes, and self-efficacy in palliative care can contribute to gaining knowledge and developing interventions to improve self-efficacy in palliative care. Thus, this study aimed to examine the moderator effect of attitudes toward ADs in the relationship between their knowledge about the care at the EOL and self-efficacy in palliative care among Korean nurses. Our hypothesis was that nurses’ attitudes moderate the relationship between knowledge and self-efficacy in palliative care.

## Methods

### Design and procedure

In a cross-sectional, correlational study, we conducted survey on a convenient sample of nurses either online or through a standardized offline procedure between March and August, 2021. Nurses who were working at four university-affiliated tertiary hospitals, a public hospital and a mental hospital in two Metropolitan cities and two large cities in South Korea were recruited using investigators’ networks. To collect data on self-efficacy in palliative care, knowledge, attitudes, and demographic and professional information, an online survey link generated using the Google form was sent to nurses who agreed to participate in the study. For the offline standardized procedure, nurse managers helped the research team screen and recruit, and collect data from nurses who agreed to participate in the study.

The Gachon University’s Bioethics Review committee approved this study (initial ethical approval code#: 1044396-202011-HR-181-01, initial approval date: December 9, 2020; amendement approval code#: 1044396-202011-HR-181-02, amendement approval date: July 7, 2021). Prior to data collection, all nurses who participated in the standard survey procedure provided a signed written informed consent. For nurses who participated in online survey, returns of online informed consent statement with his/her responses to the survey were considered as consent to participate in this study.

### Participants

Nurses were able to participate in this study if they provided bedside care at the current practice ≥ 6 months of clinical experiences in various areas, including emergency care, intensive care, hematology/hospice care, medical and surgical care, psychiatric care, and others and had an experience of at least one death of a patient in the current practice. However, nurses who suffered from serious medical conditions, such as cancer or chronic illness, were excluded because personal illness experiences might confound their professional perspectives about the EOL including ACP and ADs.

Using G-power version 3.1.9.7 [28], the sample size was computed based on a series of multiple regression analyses with two-tailed test to examine factors on self-efficacy in palliative care. When we considered the significance level of 0.05, the power of 0.80, predictors of 12 with an effect size (*f*^2^ = 0.038) from a previous research study [29], the sample size calculated was 209. Considering approximately a 70% of response rate in a prior survey study [30], an estimated sample size to recruit for this study was 299. In this study, 282 responses were used to address the aims of this study.

### Measures

Self-efficacy in palliative care was assessed using the Korean version of the 23-item Self-Efficacy in Palliative Care Scale [31], which was originally developed by Mason and colleagues in English language [32]. The original version consists of three subscales of communication (8 items), patient management (8 items), and multidisciplinary teamwork (7 items), while in the Korean and Spain versions, the patient management subscale was divided into the two factors, patient management—physical (5 items) and patient management—psychosocial-spiritual (3 items). Each item of the Korean version was constructed on a 10-point Likert scale (1 = *very anxious*; 10 = *very confident*), like the Spain version [33]. A mean score for the total was computed, with possible total scores ranging from 1 to 10, and higher scores indicating greater self-efficacy. Validity and reliability of the Spanish [33] and Korean [31] versions were previously supported. Cronbach’s alpha in this study was .965.

Knowledge about EOL care decision was assessed using the End-of-Life Care Decision Inventory, which consists of 21 items as a unidimensional scale with three response options for each item (1 = yes, 0 = no, or don’t know) [34]. The possible total scores ranged from 0 to 21, with higher scores indicating more knowledge. Validity and reliability of the scale was previously supported [34]. Kuder-Richardson coefficient in this study was .588.

Attitudes were assessed using the Korean version of the 16-item Advance Directive Attitude Survey (ADAS) [35], which was originally developed by Nolan and Bruder in English language [36]. Each item regarding an individual’s positive—negative posits for ADs was constructed on a four-point Likert scale (1 = strongly disagree; 4 = strongly agree). The possible total scores range from 16 to 64, with higher scores indicating highly positive attitudes toward ADs. Validity and reliability of the Korean version was supported [35]. Cronbach’s alpha in this study was .756.

Nurses’ demographic and professional characteristics were also collected using a standard questionnaire. Demographic characters included age, gender, marital status, religion, educational level, financial comfort, and perceived health status. Data on professional information were clinical work experience, position, education about palliative care and ADs, and patient care or personal experiences with ADs.

### Statistical analysis

The SPSS (version 26) was used for all data analyses, including descriptive and inferential statistics to address the aim and to describe sample and variable characteristics. A two-tailed test and a significance level of < .05 were used for all analyses. Descriptive statistics were used for sample description, including mean and standard deviation for continuous variables or frequency and percentage for categorical variables. Student *t*-test, Pearson’s correlation, or one-way ANOVA with post hoc test was conducted to compare the levels of self-efficacy in palliative care according to sample characteristics. Pearson’s correlation coefficients were also computed to examine association among the variables of knowledge, attitudes, and self-efficacy. Lastly, the SPSS PROCESS macro with Model 1 was used to examine the association between knowledge and self-efficacy in palliative care and the moderating effect of attitudes toward ADs in the relationship [37]. The Johnson-Neyman method and the pick-a-point method (i.e., mean minus 1standard deviation [lower positive attitudes], mean [moderately positive attitudes], mean plus 1 standard deviation [higher positive attitudes]) was performed in the PROCESS macro (version 4.1) to find out the significant transition point of moderating effect within the observed range of attitudes toward ADs.

## Results

### Participating nurses

From 439 nurses who were invited initially, 302 nurses returned with their responses (a response rate = 68.8%). After excluding duplicate responses (n = 20), a final sample was comprised of 282 nurses in palliative care (n = 44, 15.6%) and non-palliative care settings (n = 238, 84.4%), including medical-surgical (n = 70), intensive care (n = 70), emergency care (n = 59), mental healthcare settings (n = 31), and others (n = 8) (Table 1). The mean age of nurses was 30.3 years (SD = 6.1) and the majority of participants were female (90.8%) and single (74.5%). The sample predominantly had a baccalaureate education (n = 220, 78.0%). The mean work experience in current unit was 48.3 months (SD = 46.0). The majority reported no educational experience regarding ADs at work (67.7%), while the majority (63.5%) experienced patient care with ADs. Personal experiences in EOL care of relatives with ADs were found in 12.4%.

**Table 1:** Sample characteristics and differences in nurses’ self-efficacy in palliative care according to the sample characteristics (N = 282).

Characteristics	Categories	n (%) or Mean±SD	Self-efficacy in palliative care		
			Mean±SD	r *(p)*	t or F (*p*) (post-hoc test)
Age(years)	Total	30.3±6.1		.222 (< .001)	
	20–29 ^a^	168(59.6)	5.70±1.15		9.91 (< .001)
	30–39 ^b^	82(29.1)	6.09±1.19		(a<b,c)*
	≥ 40 ^c^	32(11.3)	6.67±1.40		
Gender	Female	256(90.8)	5.92±1.24		-0.36(.717)
	Male	26(9.2)	6.01±1.19		
Marital status	Single	210(74.5)	5.81±1.21		-2.67(.008)
	Married	72(25.5)	6.26±1.24		
Religion	Yes	108(38.3)	6.09±1.38		1.66(.099)
	None	174(61.7)	5.82±1.12		
Education level	Associate ^a^	25(8.9)	5.67±1.16		3.12(.046)**
	Bachelor ^b^	220(78.0)	5.88±1.14		
	≥Master ^c^	37(13.1)	6.37±1.70		
Subjective health status	Poor	13(4.6)	5.80±1.71		0.18(.838)
	Moderate	236(83.7)	5.92±1.22		
	Good	33(11.7)	6.02±1.16		
Financial comfort	Can’t make ends meet	7(2.5)	5.66±1.32		1.84(.160)
	Just enough	261(92.6)	5.90±1.24		
	Comfortable	14(5.0)	6.52±0.91		
Work experiences at current unit (months)		48.3±46.0		-.048(.418)	
Clinical areas	Palliative care setting	44(15.6)	6.28±1.15		2.08(.038)
	Non-palliative care setting	238(84.4)	5.86±1.24		
Educational experience of ADs	Yes	91(32.3)	6.21±1.17		2.74(.007)
	No	191(67.7)	5.79±1.24		
Experiences of ADs with patients	Yes	179(63.5)	6.03±1.17		1.90(.059)
	No	103(36.5)	5.74±1.32		
Personal experience for relatives with ADs	Yes	35(12.4)	6.41±1.13		2.51(.013)
	No	247(87.6)	5.86±1.23		

AD, advance directive; ER, emergency room; ICU, intensive care unit; M, mean; SD, standard deviation.

*Bonferroni test results from post-hoc.

**In post-hoc, differences were not significant.

The level of self-efficacy in palliative care significantly differed according to age, marital status, educational level, clinical areas, educational experience of ADs, and personal experience for relatives with ADs (Table 1). Younger nurses aged 20–29 years (F = 9.91; p < .001), single nurses (t = -2.67; p = .008), and lower educational levels (F = 3.12; p = .046) had lower self-efficacy than their counterparts (Table 1). Nurses who worked at the department of hematology or hospice (t = -2.08, p = .038), had educational experiences regarding ADs at work (t = -2.74, p = .007), and had personal experiences for relatives with ADs (t = -2.51, p = .013) had higher self-efficacy than their counterparts.

### Correlations among the levels of knowledge, attitudes, and self-efficacy in palliative care

The mean score of self-efficacy in palliative care was 5.92±1.23 out of 10 (Table 2). The levels of knowledge about EOL care decision (14.59±2.51 out of 21) and attitudes toward ADs (47.99±4.46 out of 64) among nurses. Knowledge, attitudes, and self-efficacy in palliative care were significantly correlated each other (Pearson’s rs = .136 - .244; all ps < .05).

**Table 2:** Correlations among the levels of knowledge, attitudes, and self-efficacy in palliative care (N = 282).

Variables	Mean ± SD (Range)	Knowledge	Attitudes
		r (*p*)	
Knowledge about EOL care decision	14.59±2.51 (4–20)	1	
Attitudes toward ADs	47.99±4.46 (37–62)	.136 (.023)	1
Self-efficacy in palliative care	5.92±1.23 (1.3–10.0)	.182(.002)	.244 (< .001)

AD, advance directive; EOL, end-of-life; SD, standard deviation.

### The relationship between knowledge and self-efficacy in palliative care and attitudes as a moderator

In the moderating model, knowledge was entered as an independent variable; attitudes as a moderator; age, marital status, clinical areas, and educational and personal experience regarding ADs as covariates, and self-efficacy in palliative care as a dependent variable (Table 3). Higher levels of knowledge (ß = 0.06, p = .048) and attitudes (ß = 0.06, p < .001), the interaction term of knowledge and attitudes (ß = 0.01, p = .025) were significantly associated with higher levels of self-efficacy in palliative care (F = 6.12, p < .001, R^2^ = .152), indicating the moderating effects of attitudes.

**Table 3:** Moderating effect of attitudes on the relationship between knowledge and self-efficacy in palliative care controlling for covariates.

Predictor	Model 1			Model 2		
	*ß*	t	*p*	*ß*	t	*p*
Constant		12.63	< .001	4.82	13.32	< .001
Age (year)	0.17	2.17	.031	0.03	1.91	.057
Marital status (Married)	0.02	0.20	.839	0.02	0.09	.925
Clinical areas (Palliative care setting)	0.08	1.35	.178	0.18	0.88	.379
Educational experience of ADs (Yes)	0.10	1.70	.090	0.25	1.62	.106
Personal experience for relatives with ADs (Yes)	0.11	1.81	.071	0.34	1.57	.118
Knowledge about EOL care decision				0.06	1.99	.048
Attitudes toward ADs				0.06	3.73	< .001
Knowledge about EOL care decision x attitudes toward ADs				0.01	2.26	.025
F(*p*)	5.05 (< .001)			6.12 (< .001)		
R^2^	.084			.152		
∆R^2^	.016			.025		

AD, advance directive; EOL, end-of-life; ∆R^2^, changes in R^2^ due to interaction term.

Reference group: Marital status, single; Clinical areas, non-palliative care setting; Educational experience of ADs, no; Personal experience for relatives with ADs, no.

**Fig 1:**
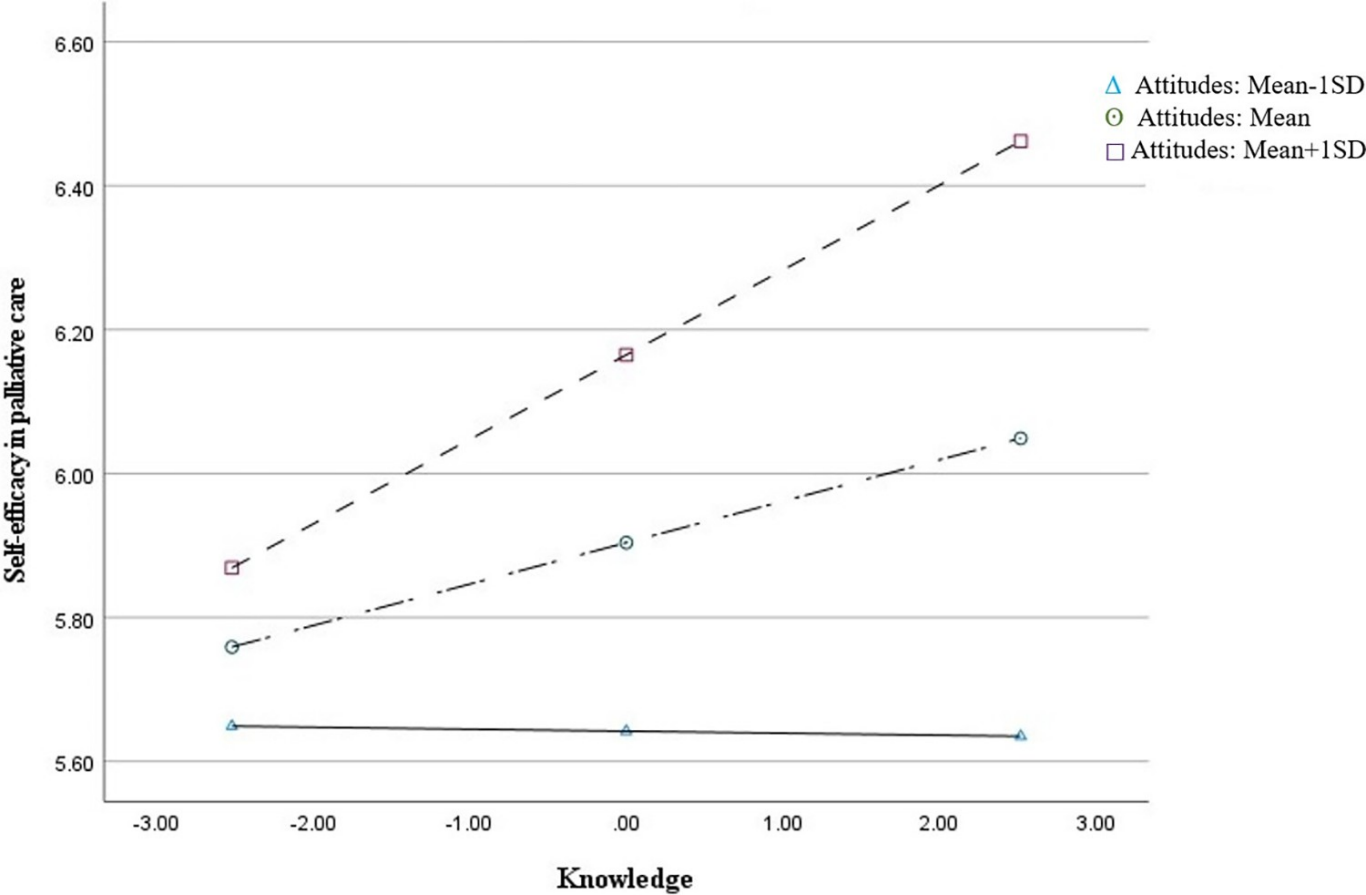
The moderating effect of attitudes toward advance directives on the relationship between knowledge about end-of-life decision and self-efficacy in palliative care.

The relationship between knowledge and self-efficacy in palliative care was increased when nurses’ attitudes toward ADs was increased, while the relationships were significant only in nurses with highly and moderately positive attitudes (R^2^ change = .016, F = 5.11, p = .025), but not nurses with lack of positive attitudes (Fig 1). The estimated effects of knowledge on self-efficacy for representative values of attitudes produced by pick-a-point approach are shown in Table 4. In specific, knowledge (about EOL decision) was significantly and positively related to self-efficacy in palliative care among nurses with moderately (ß = 0.057, p = .048) and highly positive attitudes toward advance directives (ß = 0.118, p = .006) but not significantly related to self-efficacy among those with less positive attitudes (ß = - 0.003, p = .938). Furthermore, according to the results of the Johnson-Neyman technique, specific value ranges of attitudes were identified, where a moderating effect was observed (S1 Table). Specifically, when the attitude value exceeded 47.95, the relationship between knowledge and self-efficacy was statistically significant (p < .05). However, the moderating effect was not statistically significant when attitude value was equal to or less than 47.95.

**Table 4:** Conditional effects of knowledge at values of attitudes toward ADs.

	Attitudes	Effect	SE	t	p	95% CI	
Mean-1SD	43.53	-0.003	0.04	-0.08	.938	-0.073	0.067
Mean	47.99	0.057	0.03	1.99	.048	0.001	0.114
Mean+1SD	52.46	0.118	0.04	2.76	.006	0.034	0.202

SD, standard deviation; SE, standard error; CI, confidence interval.

## Discussion

The findings of this study supported the moderating effect of nurses’ attitudes toward ADs in the relationship between knowledge about decisions of EOL care decision and their self-efficacy in palliative care. When knowledge was higher, nurses’ self-efficacy in palliative care was higher, and the relationship was further intensified in those with highly and moderately positive attitudes. The findings of this study provide knowledge on the intervention targets to improve self-efficacy in palliative care among nurses. According to the findings, not only knowledge but also attitudes need to be improved to increase self-efficacy in palliative care among nurses.

In this study, the level of self-efficacy in palliative care among Korean nurses with various clinical backgrounds, including palliative and non-palliative care settings, was moderately low based on the 0-to-100 transformed score of 55.6. Particularly, levels of self-efficacy in palliative care of nurses who were younger, single, lower educational level, and no educational and personal experiences regarding ADs were relatively low. Further, non-palliative care nurses had significantly lower levels of self-efficacy in palliative care than hematology and palliative care nurses. The results of nurses’ self-efficacy in palliative care varied by their clinical practice areas, with mixed results in prior studies. In a limited number of prior studies, oncology nurses had a low level of self-efficacy to deliver palliative care at the EOL [26], while intensive care nurses had a high level of self-efficacy [21]. On the other hand, in a sample of doctors and nurses with various clinical backgrounds who worked largely in the internal medicine, followed by the rehabilitation [24], the level of self-efficacy was moderate, with the self-efficacy of doctors and nurses in the neurology being lower than that of those in the hospice. The findings of this study add more knowledge on the levels of self-efficacy in palliative care among nurses with different clinical backgrounds, particularly low levels of self-efficacy among non-palliative care nurses. All the findings in this study and in the prior studies imply the needs for interventions targeting those nurses at early career and non-palliative care settings to improve their self-efficacy. Improvement in self-efficacy is critically important among nurses because low self-efficacy in palliative care can prevent nurses from actively engaging in EOL discussion [17, 18, 20–23, 25].

To improve self-efficacy in palliative care among nurses, determination of modifiable factors affecting self-efficacy and the mechanism in the relationships is important. Two possible modifiable factors affecting self-efficacy are knowledge and attitudes [21, 24, 26, 35, 38]. In prior studies that examined the levels of knowledge alone or attitudes together, results were inconsistent. The levels of knowledge of healthcare professionals (45% of physicians and 55% of nurses) were moderately low [39], and nurses primarily from the home care and hospice [25] and hospital nurses from the medical, surgical, and intensive care were low [40]. However, physicians and nurses for cancer patients demonstrated high levels of knowledge about ADs, while knowledge levels in nurses were considerably higher than physicians [41]. In this study, nurses’ knowledge about decisions of EOL care was moderate, based on the 0-to-100 transformed score of 69.5. On the other hand, attitudes toward ADs in this study were moderately positive, with the 0-to-100 transformed score of 66.7. This score was similar to the score (50.4) in a prior study (the transformed score of 65.0). In the prior study, the authors interpreted the score as moderately positive attitudes [36]. In other prior studies, attitudes of healthcare providers were also positive [25, 39, 40–42]. Additionally, the findings of this study show the important role of attitudes in improvements in self-efficacy in palliative care through the effects on the relationship between knowledge and self-efficacy in palliative care. Even though the levels of the mean attitudes in the total sample were moderate, some nurses had lack of positive attitudes toward ADs, and in those nurses, self-efficacy in palliative care was not increased by increased knowledge. Thus, interventions need to be provided to those nurses with lack of positive attitudes to improve attitudes, and, in turn, improve self-efficacy in palliative care.

We hypothesized that knowledge was associated with self-efficacy in palliative care, and attitudes was the moderator of the relationship, and the hypothesis was supported by the findings of this study. Knowledge [19] and/or attitudes of healthcare providers regarding ACP or ADs [43, 44] were previously documented largely associated with the likelihood of ACP practice [19, 43, 44] or performance of EOL care [38, 45]. On the other hand, their relationships to self-efficacy in palliative care have been addressed in a limited number of studies [21, 24, 26]. In the prior studies, only direct relationships of knowledge and/or attitudes to self-efficacy in palliative care have been examined. Knowledge in palliative care among oncology nurses was significantly associated with self-efficacy in palliative care [26]. In the other two studies [21, 24], higher levels of knowledge and positive attitudes or perceptions were associated with higher levels of self-efficacy among healthcare providers (doctors and nurses) and intensive care nurses. We further examined the moderator role of attitudes in the relationship between knowledge and self-efficacy, and the relationships were supported by the findings of this study. Self-efficacy in palliative care of nurses was increased by increase in knowledge only in those nurses with moderately and highly positive attitudes, but not those with lack of positive attitudes. These findings imply that increase in knowledge alone without improvement in attitudes may not sufficiently improve self-efficacy. Therefore, it is important for healthcare providers and researchers to develop and deliver interventions targeting improvements in both knowledge and attitudes, and the interventions should be tailored based on the levels of attitudes and knowledge to improve self-efficacy in palliative care.

### Study strengths and limitations

The first strength of this study is that it highlighted the levels of self-efficacy in palliative care of nurses with various backgrounds, showing the targets of interventions, including nurses who are younger and who provide non-palliative care. Another strength of this study was to show that the attitudes was a moderator in the relationship between knowledge and self-efficacy, showing the mechanism of how knowledge and attitudes improve self-efficacy. These findings demonstrate the important role of attitudes in improvement in self-efficacy in palliative care through the moderator effect. Thus, the findings of this study show two modifiable variables that can be essential components for the development of interventions to improve self-efficacy.

This study had some limitations. A cross-sectional, observational study design was used, which prevents from establishment of causal relationships among the variables and limits the generalizability of the findings of the study, requiring to warrant future studies using the cohort or experimental design. Another limitation was that a convenience sample of nurses recruited from hospitals in large urban cities, not rural areas, may also limit the generalizability of the findings of the study.

## Conclusion

Nurses’ higher levels of knowledge and more positive attitudes, and the interaction term of knowledge and attitudes in nurses were significantly associated with higher levels of self-efficacy in palliative care, indicating the moderator role of attitudes. The relationship between higher levels of knowledge and higher levels of self-efficacy in palliative care was significant only in nurses with moderately and highly positive attitudes, but not nurses with lack of positive attitudes. To improve self-efficacy in palliative care among nurses, efforts should be needed to enhance not only their knowledge but also their attitudes.

### Implications for practice and research

The results of this study provide basic data on EOL care issues from nurses with various clinical background who are relatively isolated in such type of care. Our results show the needs for interventions targeting improvements in nurses’ attitudes toward ADs and knowledge to increase their self-efficacy in palliative care. In a previous study [46], one palliative care education for four hours based on didactic education, discussion and small group exchanges, and case studies improved knowledge and attitudes in palliative care among oncology nurses. The specific content of the education included symptom management, including pain, the definition of palliative care, and communication strategies [46]. The findings imply that knowledge and attitudes can be improved by educational interventions. Further research is warranted to examine whether improvements in knowledge and attitudes improve self-efficacy in palliative care among nurses using well-designed interventions, such as randomized controlled trials.

## Supporting information

S1 Table The range of attitudes toward advance directives in the relationship between knowledge and self-efficacy in palliative care. (PDF)

S1 Dataset Study dataset. (XLSX)
