# Supplementary material for: The moderating effect of attitudes in the relationship between knowledge and self-efficacy in palliative care among nurses: A cross-sectional, correlational study
Source: PLoS One. 2023 Oct 5;18(10):e0292135. doi: 10.1371/journal.pone.0292135 (PMC10553266; doi:10.1371/journal.pone.0292135)
Supplement: S1 Table — (PDF) [file pone.0292135.s001.pdf]

S1 Table. The Range of Attitudes toward Advance Directives in the Relationship between Knowledge and Self-efficacy in Palliative Care

Attitudes	Effect	SE	t	p	95% CI	
37.00	-0.09	0.07	-1.36	.175	-0.223	0.041
38.25	-0.07	0.06	-1.23	.220	-0.193	0.044
39.50	-0.06	0.05	-1.06	.288	-0.163	0.049
40.75	-0.04	0.05	-0.85	.398	-0.134	0.053
42.00	-0.02	0.04	-0.56	.576	-0.106	0.059
43.25	-0.01	0.04	-0.18	.858	-0.079	0.066
44.50	0.01	0.03	0.32	.752	-0.054	0.074
45.75	0.03	0.03	0.92	.360	-0.031	0.085
47.00	0.04	0.03	1.55	.123	-0.012	0.100
47.95	0.06	0.03	1.97	.050	0.000	0.114
**48.25**	**0.06**	**0.03**	**2.09**	**.038**	**0.003**	**0.118**
**49.50**	**0.08**	**0.03**	**2.45**	**.015**	**0.015**	**0.140**
**50.75**	**0.10**	**0.04**	**2.65**	**.009**	**0.024**	**0.165**
**52.00**	**0.11**	**0.04**	**2.74**	**.006**	**0.031**	**0.192**
**53.25**	**0.13**	**0.05**	**2.77**	**.006**	**0.037**	**0.220**
**54.50**	**0.15**	**0.05**	**2.77**	**.006**	**0.042**	**0.248**
**55.75**	**0.16**	**0.06**	**2.76**	**.006**	**0.046**	**0.278**
**57.00**	**0.18**	**0.07**	**2.74**	**.007**	**0.050**	**0.308**
**58.25**	**0.20**	**0.07**	**2.71**	**.007**	**0.054**	**0.338**
**59.50**	**0.21**	**0.08**	**2.69**	**.008**	**0.057**	**0.368**
**60.75**	**0.23**	**0.09**	**2.67**	**.008**	**0.060**	**0.399**
**62.00**	**0.25**	**0.09**	**2.64**	**.009**	**0.063**	**0.430**

SE, standard error; CI, confidence interval.

Bold indicates statistical significance.
